# A DSSM network for inferring and prioritizing cell-type-specific regulons using single-cell RNA-seq data

**DOI:** 10.1186/s12859-025-06329-4

**Published:** 2025-12-07

**Authors:** Yaxin Fan, Yichao Mei, Shengbao Bao, Jianyong Wang, Junxiang Gao

**Affiliations:** Hubei Key Laboratory of Agricultural Bioinformatics, College of Informatics, Huazhong Agricultural University, Wuhan, 430070 China

**Keywords:** Regulon, Deep structured semantic model, scRNA-seq, Cell type specificity, Deep learning

## Abstract

**Background:**

Transcription factors and their target genes form regulatory modules known as regulons, which exhibit significant specificity across various cell types. The integration of single-cell transcriptome data, transcription factor motif data, and ChIP-seq data presents a challenging task in identifying cell-type-specific regulons and examining their activities.

**Results:**

In response, this study presents a Deep Structured Semantic Model for inferring and prioritizing cell-type-specific Regulons (DSSMReg). This approach utilizes single-cell transcriptome and transcription factor motif data to map transcription factors and target genes into a low-dimensional semantic space, resulting in the generation of feature vectors. The model then computes the cosine similarity between transcription factors and target genes to evaluate their regulatory strength and subsequently infers cell-type-specific regulons based on this assessment. Moreover, DSSMReg employs the AUCell algorithm to rank the importance of regulons for each cell type.

**Conclusions:**

We compared DSSMReg against five representative gene regulatory inference algorithms using scRNA-seq data from five cell lines, with DSSMReg achieving the highest evaluation metrics for both AUROC and AUPRC. Furthermore, we applied DSSMReg to infer cell-type-specific regulons from scRNA-seq data of triple-negative breast cancer and human bone marrow hematopoietic stem cells. Our results indicated that regulons with high AUCell scores possess significant biological relevance. The source code of DSSMReg is freely available at https://github.com/YaxinF/DSSMReg.

**Supplementary Information:**

The online version contains supplementary material available at 10.1186/s12859-025-06329-4.

## Background

In eukaryotes, a transcription factor (TF) recognizes specific sites within the regulatory regions of target genes (TGs) and bind to DNA sequences to activate or suppress gene expression. A gene module composed of a TF and its regulated TGs is referred to as a regulon. Regulons can coordinate gene expression, synchronously activating or repressing a set of genes that have related functions or are involved in the same biological process, in response to changes in the intracellular or extracellular environment, thereby fulfilling specific biological functions [1, 2]. Regulons exhibit varying effects across different cell types, displaying significant specificity that is essential for biological processes, including cell differentiation, development, metabolism, and pathogenesis [3, 4].

Traditional bulk RNA-seq lacks the ability to reveal differences between distinct cell types. Single-cell RNA sequencing (scRNA-seq) significantly improves our understanding of the heterogeneity of different cell types and facilitates the inference of cell-type-specific regulatory mechanisms [5, 6]. Cell-type-specific regulation is crucial for biological processes, supporting the specificity of cell differentiation and function. One widely used idea for inferring gene regulation from expression data is the construction of co-expression networks based on correlation [7, 8]. However, these methods face challenges in distinguishing between direct and indirect relationships, which can lead to a considerable number of false positives [9]. The interaction of transcription factors with DNA motifs influences the spatiotemporal expression patterns of genes. Integrating DNA motif with single-cell gene expression data can help mitigate the issue of false positives associated with co-expression-based inference, thereby improving the accuracy of gene regulatory predictions.

Multiple methods have been employed to infer gene regulation from single-cell transcriptome sequencing data [10–12]. Among these, GENIE3 and SCENIC [13] are recognized as two prominent classic algorithms. GENIE3 addresses the prediction challenge by decomposing it into multiple regression tasks. For each TG, the algorithm considers all other genes as potential regulatory factors, striving to establish a regression model utilizing random forests to predict the expression levels of the target genes. While originally designed for bulk RNA-seq data, GENIE3 has exhibited lower predictive accuracy when applied to single-cell datasets. SCENIC builds upon GENIE3 by incorporating cis-regulatory motif analysis. It first calculates co-expression relationships, subsequently filtering out false positives through the use of transcription factor motifs to delineate regulatory relationships between TFs and their TGs. The introduction of various deep learning models has further advanced this field. One such model is DeepSEM, which is structured upon the β-VAE framework. This model infers regulatory relationships among genes by constructing a neural network-based structural equation model (SEM) and has demonstrated good performance across multiple benchmark datasets [14]. Additionally, the MetaSEM model comprises three components: an encoder, a meta-decoder, and a gene regulatory network (GRN) layer. The encoder transforms high-dimensional single-cell gene expression data into feature vectors, while the meta-decoder models the regulatory relationships. The GRN layer employs a structural equation model to infer gene regulatory relationships, thereby guiding the training of the meta-decoder [15]. The DeepRIG model computes the Spearman correlation coefficients between gene pairs in single-cell RNA sequencing data, constructing a weighted gene co-expression network. Subsequently, global regulatory signals are extracted from the weighted gene co-expression network using a graph autoencoder, which is then utilized to reconstruct the gene regulatory network [16].

The deep structured semantic model (DSSM), initially developed for recommendation systems, has exhibited superior performance in predicting links between pairs of entities [17, 18]. This efficiency can be attributed to its distinctive structural design and robust feature extraction capabilities. The DSSM consists of two independent substructures, commonly referred to as the “two towers”. Each tower is responsible for extracting key features of the entities and converting them into low-dimensional vector representations, which are subsequently utilized to calculate the degree of association between the entities. For instance, in the context of recommendation systems, the user tower is designed to represent user characteristics, encompassing historical behaviors, preference interests, and statistical information. Conversely, the item tower focuses on representing item features, such as attributes, categories, and interaction history with users. The primary objective of the DSSM is to ascertain the degree of association between users and items, thereby facilitating the generation of recommendations. This model effectively accommodates multiple features for varying types of entities, irrespective of their similarities or differences. Furthermore, it supports the design of diverse network architectures tailored to specific tasks, which enhances the flexibility and accuracy of the recommendation process. The applicability of the deep structured semantic model extends into certain biological research domains. For example, researchers have developed DeepPPI, a method that leverages the composition, distribution, and sequence order of amino acids, to derive protein representations. These representations of two proteins are then input into two independent neural networks to predict protein–protein interactions, with DeepPPI demonstrating commendable performance across multiple datasets [19]. In another instance, Wan et al. introduced DeepCPI, which utilizes unsupervised representation learning methodologies, such as latent semantic analysis and Word2Vec, to generate low-dimensional representations of compound and protein features. These representations are subsequently fed into two independent multimodal neural networks to predict compound-protein interactions [20]. Additionally, a recent study employed a deep structured semantic model, processing two sets of infrared spectral data through deep neural networks to train paired spectra for the detection of ductal carcinoma in situ [21]. These examples collectively illustrate the broad applicability of the deep structured semantic model. In contexts where the link prediction between two distinct entities is required, the DSSM presents a promising framework, provided that the features of the entities can be accurately represented as vectors. In the present study, both TFs and TGs may be characterized through the analysis of scRNA-seq data and motif data. Accordingly, employing a deep structured semantic model to map TFs and TGs into a low-dimensional semantic space to predict their regulatory relationships constitutes a sound algorithmic framework.

We introduce DSSMReg, a model that integrates scRNA-seq data with TF motif data to identify key regulons across different cell types. Built on a deep structured semantic model framework, DSSMReg employs both an autoencoder and a skip-gram model to generate feature vectors for TFs and TGs obtained from the scRNA-seq and motif data. DSSMReg utilizes publicly available gene interaction data to train the model, enabling it to infer cell-type-specific regulons effectively. To assess the model’s performance, we compared DSSMReg with five other algorithms, and our results clearly indicate that it outperformed the others in both Area Under the Receiver Operating Characteristic (AUROC) and Area Under the Precision-Recall Curve (AUPRC). Furthermore, we applied DSSMReg to infer cell-type-specific regulons from scRNA-seq data related to triple-negative breast cancer (TNBC). The results revealed that the inferred regulons were enriched with known cell-type-specific marker genes. Notably, among the highly ranked regulons in malignant tumor cells, we identified 12 genes closely linked to the onset, progression, and suppression of triple-negative breast cancer. This indicates that the regulons recognized by DSSMReg have specific biological correlations. Highly active TF or cancer-related genes may be valuable in drug target screening. Additionally, we utilized DSSMReg on a dataset of scRNA-seq data from human bone marrow hematopoietic stem cells. The findings demonstrate that the model can proficiently infer key genes at various differentiation stages, highlighting its potential in exploring the biological processes underlying cell differentiation.

## Results

### The framework of DSSMReg

**Fig. 1:**
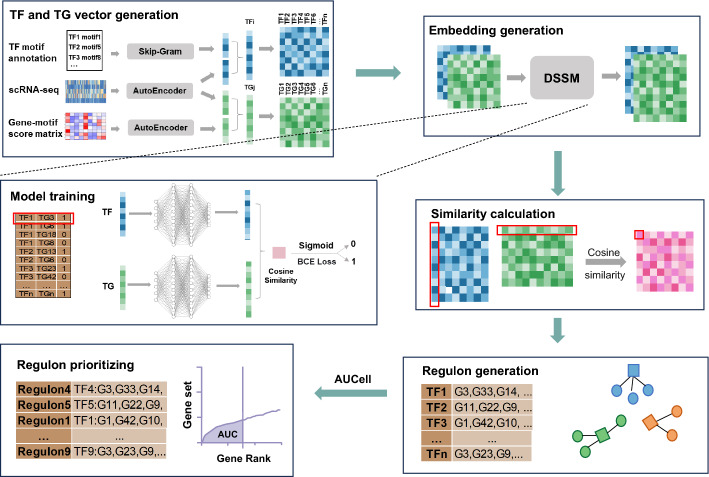
Workflow of DSSMReg. DSSMReg first generates input vectors for the deep structured semantic model for each transcription factor and potential target gene using the gene-motif score matrix, transcription factor motif annotations, and scRNA-seq data. Subsequently, DSSMReg trains the model using cell-type-specific regulatory relationships validated by ChIP-seq experiments, producing vectors for transcription factors and target genes. The model then calculates the cosine similarity between the vectors of transcription factors and target genes, selecting corresponding target genes for each transcription factor based on the scores to form a regulon. Finally, DSSMReg utilizes the AUCell tool to calculate and rank the scores of each regulon within the cells

Initially, we generate feature vectors for TFs and TGs using scRNA-seq data and motif data (Fig. 1). The scRNA-seq data undergoes preprocessing and normalization, resulting in a single-cell gene expression matrix where rows correspond to genes, columns correspond to individual cells, and the elements indicate the levels of gene expression. To analyze this data, we constructed a standard autoencoder designed to encode the expression vectors of each gene derived from the scRNA-seq dataset. The autoencoder consists of two primary components: the encoder and the decoder. During training, the encoder transforms the input data into a lower-dimensional representation, while the decoder reconstructs the original data from this representation, striving to ensure that the reconstructed output closely matches the input. This lower-dimensional representation effectively captures the essential information and relationships present within the expression data. After training is completed, the single-cell gene expression matrix serves as input for the standard autoencoder model. The low-dimensional representations generated by this model subsequently function as the expression feature vectors for TFs and TGs.

TF motif annotations offer valuable insights into the binding patterns between TFs and specific DNA sequences. We obtained TF motif annotation data from the CisTarget database [13] and employed the skip-gram model from Word2Vec to generate feature vectors for these motifs. The central idea of the skip-gram model is to learn the vector representation of a central word by predicting the context words that surround it. In this context, we consider the TF as the “central word” and the motifs annotated for that TF as the “context”. This approach allows us to derive motif vectors for transcription factors [22]. We then concatenate the TF’s expression vector with its motif vector to create a composite vector that encapsulates the features of the transcription factor.

The motifs located in the regulatory regions of TGs are critical to gene expression. The CisTarget database provides a gene-motif score matrix that quantifies the strength of the relationship between motifs and genes [13]. In this matrix, the rows correspond to genes, while the columns correspond to motifs. Each element within the matrix indicates the scores of cis-regulatory modules for gene sequences situated 500 bp upstream and 100 bp downstream of the transcription start site. An autoencoder was constructed utilizing the gene-motif score matrix as input, while retaining the same structure and parameters (as detailed in the Materials and Methods section). Upon completion of the training process, the low-dimensional representations generated by the autoencoder serve as motif vectors for the target genes. The expression vector of each TG is concatenated with its corresponding motif vector to create a composite vector that encapsulates the features of the TG. At this point, feature vectors for all TFs and TGs have been obtained, which will be employed for training the deep structured semantic model and predicting gene regulatory relationships. The deep structured semantic model is comprised of two independent deep neural networks (DNNs) that share a common architecture, consisting of three fully connected layers. The validation of cell-type-specific TF-TG regulatory relationships, conducted through ChIP-seq experiments, serves as the basis for model training and performance evaluation. Once the model training is finalized, it produces embedded representations of all TFs and TGs. Subsequently, the cosine similarities between the embedded representations of each TF and its corresponding TGs are computed, yielding a regulatory score. This similarity scores serve to evaluate the regulatory strengths between TFs and TGs, whereby a higher score indicates an increased likelihood of a regulatory relationship. The identification of the TGs regulated by TFs for each cell type is based on the regulatory scores obtained from prior steps. The TF and its TGs collectively constitute a regulon. From the above description, it is evident that cell-type specificity is embedded in DSSMReg’s inference process. Although the model is trained on non-specific ChIP-seq data to learn basic regulatory relationships, during inference, we independently generate specific gene expression feature vectors for each annotated cell type using the cell type annotations from the original research as model inputs. Independent generation of cell-type-specific vectors means that the same gene has distinct vector representations across cell types, allowing the trained model to produce distinct regulatory network predictions for each cell type’s specific expression environment.

To evaluate the biological significance and functionality of regulons across various cell types, we utilized the AUCell method [13] to rank the importance of each regulon. This method assesses the activation of each gene set based on its expression levels in single-cell transcriptome data. By employing this approach, we can quantitatively analyze the functionality of different regulons across cell types and establish an objective ranking of their significance. As a result, we identified regulons that exhibit high activity in specific cell types, which provides insight into their potential biological functions and regulatory mechanisms.

During model training, we set the number of epochs to 200 without employing early stopping. The number of genes primarily determines the model’s resource usage and computational complexity, and the time and memory consumption for different gene numbers are shown in Supplementary Table S1. All experiments were conducted on the RTX3090 GPU, which has 10,496 CUDA cores and a graphics memory capacity of 24 GB.

### Comparison with existing methods

This study evaluates the performance of DSSMReg in inferring regulatory relationships by utilizing the scRNA-seq dataset obtained from BEELINE, alongside ChIP-seq validated regulatory relationships across five distinct cell types: human embryonic stem cells (hESC), human hepatocytes (hHEP), and mouse hematopoietic stem cells (mHSCs) differentiated into three lineages: erythroid (E), lymphoid (L), and granulocyte–macrophage (GM). The original study provided the annotations for these cell types [23]. DSSMReg was compared with five established GRN inference methods: DeepRIG [16], MetaSEM [15], DeepSEM [14], GENIE3 [8], and PIDC [12]. These methods have demonstrated notable performance and encompass a range of operational principles. DeepSEM employs a β-VAE framework to conjointly model GRNs and scRNA-seq data through structural equation modeling, while MetaSEM utilizes a meta-learning framework for GRN inference to elucidate gene regulation. DeepRIG integrates a weighted gene co-expression network with a graph autoencoder to infer the global regulation of genes. GENIE3 operates as a tree-based ensemble machine learning method, while PIDC is grounded in multivariate information theory for GRN inference.

**Fig. 2:**
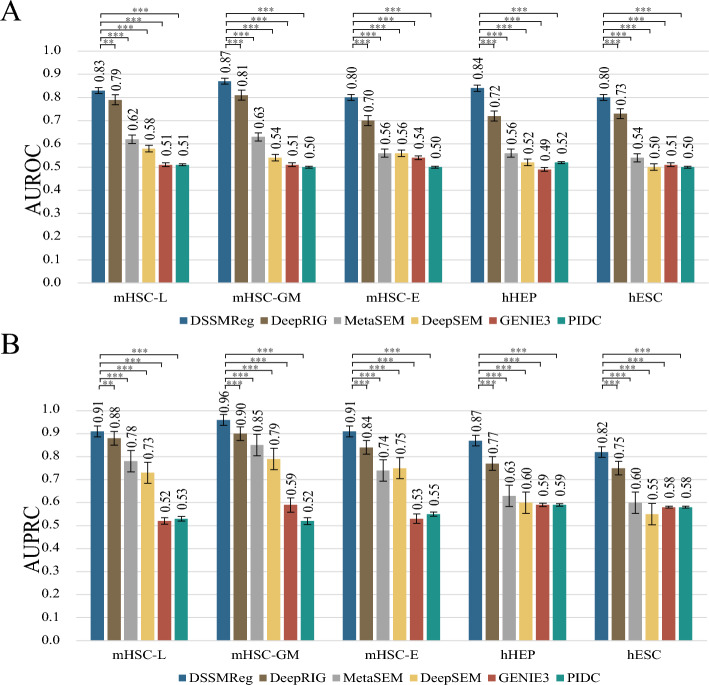
Performance comparison of DSSMReg with existing algorithms on scRNA-seq data from five cell lines. **A** Comparison of AUROC metrics. Statistical significance was determined using paired *t*-test (*p < 0.05, **p < 0.01, ***p < 0.001). **B** Comparison of AUPRC metrics

The performance of the aforementioned methods was assessed using AUROC and AUPRC. Among the five cell types analyzed, DSSMReg achieved superior performance, obtaining the highest AUROC and AUPRC values of 0.84 and 0.89, respectively (Fig. 2A, B). The second-best method, DeepRIG, recorded average AUROC and AUPRC values of 0.76 and 0.83, respectively. Overall, DSSMReg outperformed all five baseline methods in both AUROC and AUPRC, affirming that this approach effectively extracts underlying patterns from gene expression and motif information while leveraging known regulatory relationships to identify potential ones.

### DSSMReg demonstrates robustness to variations in cell number

**Fig. 3:**
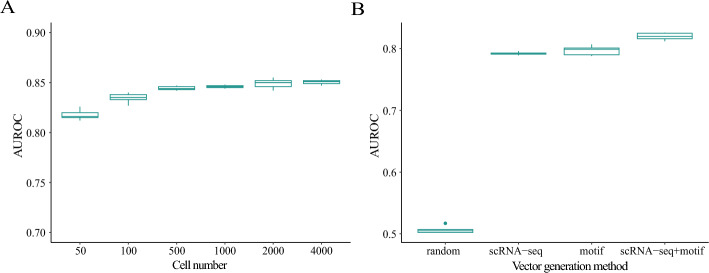
Effect of cell numbers and data types on model performance. **A** Effect of varying cell numbers on model performance. **B** Effect of different data types on model performance

The number of cells captured during single-cell transcriptome sequencing can vary significantly across samples. High-throughput technologies can process hundreds to tens of thousands of cells per experiment, making them suitable for large-scale analyses. SMART-seq methods, due to their plate-based approach, typically process dozens to hundreds of cells, offering higher sensitivity and transcript coverage that make them valuable for studies requiring detailed analysis of individual cells despite their lower throughput. Moreover, even when utilizing the same technology, the number of cells captured can fluctuate considerably. The ability to predict regulatory relationships based on varying cell numbers holds important practical implications. To investigate the effect of cell number on the performance of DSSMReg, a dataset of triple-negative breast cancer tumor cells with a high initial cell count was downsampled. Regulatory relationships were inferred from the downsampled subsets, and the AUROC was calculated to assess how DSSMReg’s performance is influenced by differing cell numbers. Given that the dimensionality of the autoencoder’s hidden vectors is set to 50, a minimum of 50 cells was sampled to accommodate the requirements of most scenarios. Six subsets were generated by sampling 50, 100, 500, 1000, 2000, and 4000 cells. The results revealed that the AUROC consistently increased with the number of cells (Fig. 3A). It stabilized once the cell number exceeded 2000. While increasing the cell number from 500 to 2000 resulted in only a slight improvement in model performance, decreasing the cell number from 500 to 50 led to a modest decline in AUROC. Nevertheless, even with as few as 50 cells, DSSMReg maintained satisfactory performance, achieving an AUROC value of 0.82. These experimental findings indicate that DSSMReg is not sensitive to fluctuations in cell numbers and can effectively infer regulatory relationships using expression data from a minimum of 50 cells. This characteristic enhances the applicability of DSSMReg across a variety of experimental contexts.

### Both scRNA-seq data and motif data enhance the model’s performance

The scRNA-seq data provides insights into gene expression levels, while the motif data comprises regulatory elements that influence these levels by modulating the binding of transcription factors. Our goal was to investigate the individual contributions of scRNA-seq data and motif data to the effectiveness of DSSMReg. The acquisition of motif data through experimental methods requires precise design and execution, which can sometimes lead to the unavailability of such data. Consequently, the ability of DSSMReg to predict regulatory relationships in the absence of motif data is a vital consideration. To address this challenge, we conducted experiments utilizing a human embryonic stem cell dataset. We generated gene embeddings through four distinct approaches: randomly generated gene generation, using only scRNA-seq data, using only motif data, and combining both scRNA-seq and motif data. The results demonstrated that DSSMReg could effectively predict regulatory relationships with just scRNA-seq data, achieving an AUROC of 0.79. In contrast, the AUROC was approximately 0.5 when using randomly generated gene vectors, indicating performance akin to random guessing. We randomly generated the gene vector. To ensure fairness in comparison, the dimensions of the random vectors in our model are consistent with those generated by DSSMReg, which are 100 dimensions. Each dimension of the random vector is independently sampled from a uniform distribution *U* (0,1). When utilizing only motif data, the performance was comparable to that of using only scRNA-seq data. However, integrating both scRNA-seq and motif data elicited a notable improvement in model performance, resulting in an AUROC of 0.83 (Fig. 3B). These findings illustrate that the autoencoder adeptly extracts and integrates TF regulatory information from both gene expression data and motif data. Therefore, when both data types are accessible, it is advisable to use them as inputs for DSSMReg to maximize predictive performance.

### DSSMReg is capable of identifying cell-type-specific regulons

**Fig. 4:**
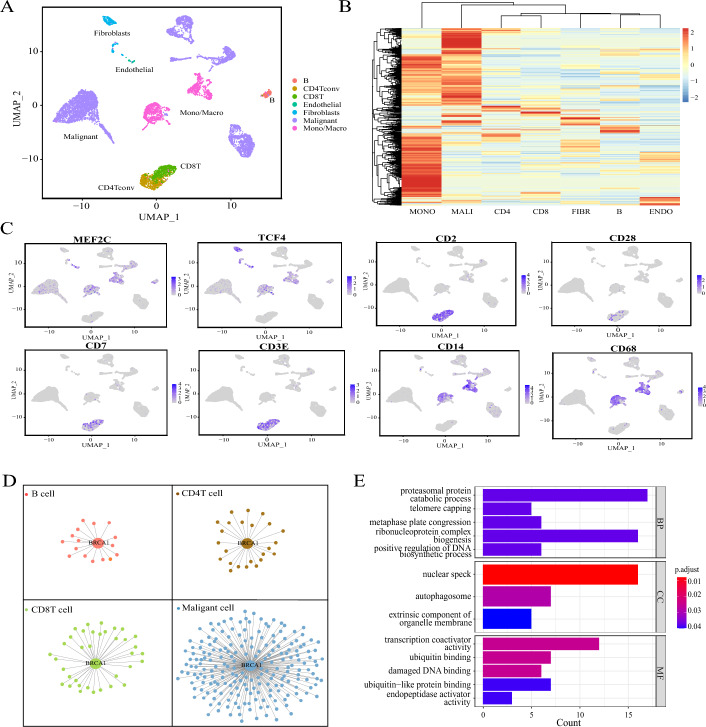
Inference of cell-type-specific regulons by DSSMReg. **A** UMAP visualization of scRNA-seq data from TNBC patients. **B** Normalized clustering heatmap of target gene counts in regulons across seven cell types. **C** Specific expression of eight marker genes (*CD2*, *CD28*, *CD7*, *CD3E*, *CD14*, *CD68*, *TCF4*, and *MEF2C*) in the seven cell types. **D** The *BRCA1* regulon exhibits a complex regulatory network in malignant tumor cells, while only a simple network is present in other cell types. **E**. GO enrichment analysis of the *BRCA1* regulon in malignant tumor cell types

To assess the capability of DSSMReg in inferring cell-type-specific regulons, we applied it to single-cell RNA sequencing data derived from patients with triple-negative breast cancer [24]. We acquired scRNA-seq data from tumor tissues of five untreated primary TNBC patients via the TISCH2 database [25], which provides comprehensive cell type annotations. Following rigorous quality control and filtering processes, we obtained expression data for 8,890 cells across 19,356 genes. These cells were classified into seven distinct cell types: B cells, CD4 T cells, CD8 T cells, endothelial cells, fibroblasts, malignant tumor cells, and monocytes-macrophages, as illustrated in Fig. 4A. The ground truth for model training consisted of 154,317 regulatory relationships within breast tissue, sourced from the Cistrome database [26] and validated by ChIP-seq experiments. DSSMReg computed TF-TG regulatory scores for each cell type, retaining only those regulatory relationships with scores exceeding 0.9. Each transcription factor, along with its corresponding target genes, constituted a regulon, enabling the inference of the respective regulon for each cell type. We detailed the number of TFs, TGs, and TF-TG regulatory relationships in the gene regulatory network of each cell type (Supplementary Table S2), with the number of TFs also indicating the number of regulons. The results indicated that regulons exhibited significant specificity to cell types, with considerable variation in the number of regulons across different cell types. Even for identical TFs, TGs varied markedly among the various cell types. Figure 4B displays a normalized heatmap depicting the number of TGs in each regulon, whereby the quantity of TGs reflects the regulatory activity level of the regulon. The observed disparities in expression and activity of these regulons across diverse cell types underscore the substantial variability in transcriptional regulation within the tumor microenvironment. These findings demonstrate that DSSMReg is proficient in inferring cell-type-specific regulons.

Marker genes exhibit distinctive expression patterns across various cell types, making them valuable for identifying, classifying, and characterizing these cell types. The Cellmarker2 database [27] offers a comprehensive resource that compiles information on 1,715 human cell types and 15,737 cell markers. Our analysis revealed that many of the marker genes identified in this database were predicted by DSSMReg. In Fig. 4C, we present eight of these markers as examples, illustrating their significant cell type specificity in expression. For B cells, we examined the regulons of *MEF2C* and *TCF4*, both of which are recognized as marker genes for this cell type. Importantly, *MEF2C* is a TF that regulates calcineurin in B cells and is essential for their proliferation [28]. Further exploration of the genes regulated by *MEF2C* led us to identify another B cell marker gene, *VIM*. In the context of CD4 T cells, we discovered the TGs of *RUFY3*, which included four T cell marker genes: *CD2*, *CD28*, *CD7*, and *CD3E*. Additionally, in the regulons associated with macrophages, we identified two macrophage marker genes, *CD14* and *CD68*, among the TGs of *CEBPD* and *EZH2*, respectively. These marker genes displayed elevated expression levels in their corresponding cell types (Fig. 4C). This outcome indicates that the regulons inferred by DSSMReg contain marker genes that are specific to certain cell types.

### Prioritizing the activity of cell-type-specific regulons

Prioritizing the activity of regulons is crucial for gaining insights into the influence and significance of specific TFs across different cell types and conditions. For instance, in cancer research, identifying TFs that play critical roles in particular biological processes or pathological states can illuminate which factors substantially affect tumor growth and progression. By recognizing highly active regulons, researchers can also identify which regulatory networks are activated under specific conditions, revealing the intricate relationships among genes. AUCell is an effective tool for assessing the activity of gene sets within single-cell RNA sequencing data [13]. It is especially valuable for investigating the activity of specific biological processes or signaling pathways within single-cell datasets.

We employed AUCell to assess the activity of regulons across various cell types. In cancerous tissues, malignant tumor cells are identified as the most detrimental cell type. Consequently, we focused on the top 20 ranked regulons predicted by DSSMReg specifically in these malignant tumor cells. Among these, 12 have been documented as associated with triple-negative breast cancer or breast cancer in general, including *HSPA1L* [29], *H2AFZ* [30], *ID1* [31], *EZR* [32], *ERF* [33], *E4F1* [34], *EZH2* [35], *PML* [36], *MBD2* [37], *PAXIP1* [38], *BRCA1* [39], and *CPSF4* [40]. Furthermore, three regulons, while lacking direct evidence linking them to breast cancer, have demonstrated significant roles in other cancer types. These include *TOB2* [41], *UBP1* [42], and *ZCCHC14* [43]. The remaining five regulons merit additional investigation. Collectively, these findings suggest that the cell-type-specific regulons identified by DSSMReg, particularly those with high rankings, hold substantial biological relevance, indicating that highly active TFs or cancer-related genes could serve as valuable drug targets.

Approximately 70% of breast cancer cases in women are associated with mutations in the *BRCA* gene family, particularly *BRCA1*, which serves as a susceptibility gene involved in DNA damage repair pathways. Notably, around 60% of breast cancers linked to *BRCA1* mutations present as triple-negative breast cancer [39]. In light of the critical importance of the *BRCA1* gene in this specific subtype of cancer, we undertook an in-depth investigation of the *BRCA1* regulon. Our analysis indicated that the *BRCA1* regulon in malignant tumor cells comprises a greater number of TGs and exhibits a more intricate regulatory network than those found in various immune cell types, which display simpler networks (Fig. 4D; Supplementary Table S3). This complexity is consistent with *BRCA1*’s substantial biological functions in cancer. Subsequently, we conducted Gene Ontology (GO) enrichment analysis on the gene set comprising the *BRCA1* regulon in tumor cells. The results demonstrated that this regulon is significantly enriched in positive regulatory processes related to DNA biosynthesis and biological processes associated with proteasome-mediated protein degradation. Additionally, the *BRCA1* regulon gene set was significantly enriched for molecular functions pertaining to damaged DNA binding, ubiquitin binding, ubiquitin-like protein binding, and endopeptidase activator activity (Fig. 4E). These findings align with prior experimental research demonstrating that *BRCA1* is integral to nucleotide excision repair and various other critical biological processes, including DNA repair, cell cycle checkpoints, apoptosis, and proteasome-mediated protein degradation [44]. *BRCA1* operates as an E3 ubiquitin ligase, with the *BRCA1-BARD1* complex localizing to damaged chromatin following DNA replication and facilitating the ubiquitination of histone H2A and additional cellular targets. Dysregulation of the ubiquitination system can result in excessive protein stabilization or impaired degradation, thereby fostering tumorigenesis [45]. These observations suggest that DSSMReg possesses the capability to identify TFs and TGs closely associated with disease progression and therapeutic responses through the inference of regulons.

### DSSMReg infers regulons in human hematopoietic stem cell differentiation

**Fig. 5:**
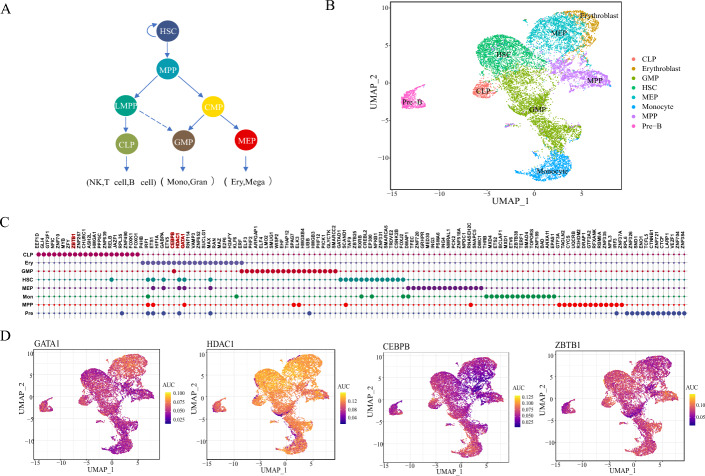
Inference of regulons in hematopoietic stem cell differentiation by DSSMReg. **A** Schematic representation of human hematopoietic cell lineage differentiation. **B** UMAP visualization of scRNA-seq data from eight cell types. **C** Top 20 regulons ranked for each cell lineage. **D** AUCell activity of the regulons predicted by DSSMReg across different cell types

Cell differentiation is a complex and systematically regulated process that involves multi-layered regulatory networks, including gene expression, transcription factor interactions, and signal transduction. In our study, we employed human bone marrow hematopoietic stem cells as a model to explore whether DSSMReg can identify regulons that play critical roles in cell differentiation. Human bone marrow contains hematopoietic stem cells (HSC), which can develop into multipotent progenitor cells (MPP). These MPPs can further differentiate into lymphoid-primed multipotent progenitors (LMPP) and common myeloid progenitors (CMP). LMPPs give rise to common lymphoid progenitors (CLP), which subsequently mature into T cells, B cells, and natural killer (NK) cells. In contrast, CMPs differentiate into granulocyte-monocyte progenitors (GMP) and megakaryocyte-erythroid progenitors (MEP). GMPs then develop into monocytes and granulocytes, while MEPs further differentiate into erythroblast and megakaryocytes [46] (Fig. 5A). To assess the role of DSSMReg in cell fate differentiation, we applied it to a scRNA-seq dataset of human bone marrow hematopoietic stem cells [47]. Figure 5B illustrates a UMAP plot of the scRNA-seq dataset, which encompasses eight distinct cell types: HSC, MPP, GMP, MEP, CLP, precursor B cells (Pre-B), monocytes, and erythroblasts. Utilizing DSSMReg, we inferred the regulatory relationships between TFs and TGs for each cell type, selecting the top 200 TGs with the highest scores for each TF to construct a regulon.

We employed AUCell to prioritize the activity of regulons for each cell type. Figure 5C presents the top 20 ranked regulons, underscoring those that fulfill significant functions within their respective cellular contexts. Among these, *GATA1* and *HDAC1* are notably prominent in both megakaryocyte-erythroid progenitors and erythroblasts. *GATA1* is a critical factor in erythropoiesis, binding to specific DNA sequences to activate genes related to erythrocyte differentiation [48]. It facilitates the transcription of α- and β-globin genes, which encode essential components of hemoglobin, thereby enhancing the erythrocytes’ oxygen-carrying capacity [49]. Figure 5D illustrates the AUCell activity of four TFs—*GATA1*, *HDAC1*, *CEBPB*, and *ZBTB1*—demonstrating a correlation between the predicted activity of these regulons and their respective functions in specific cell types. *GATA1* exhibits elevated AUCell scores in both megakaryocyte-erythroid progenitors and erythroblasts. *HDAC1* serves as a pivotal regulatory factor modulating the primary TFs involved in erythropoiesis. It interacts with all essential TFs for this biological process, activating *GATA1* and inhibiting PU.1 [50]. Moreover, *HDAC1* is integral to maintaining the homeostasis of HSCs, where it also demonstrates considerable activity [51] (Fig. 5D). *CEBPB* ranks among the top regulons for GMPs and plays a vital role in monocyte formation [48] (Fig. 5D). *ZBTB1* ranks eighth among CLPs, exhibiting a decrease in expression during bone marrow differentiation, while its levels remain stable during lymphoid differentiation. This regulation of *ZBTB1* expression prevents the activation of the typical myeloid program in LMPP cells, thereby ensuring the production of lymphocytes [52] (Fig. 5D). These findings indicate that DSSMReg is capable of effectively investigating the differentiation of hematopoietic stem cells into various blood cell types, elucidating key genes and regulatory networks at distinct stages of differentiation.

### DSSMReg predicts the dynamic changes in cells during differentiation

**Fig. 6:**
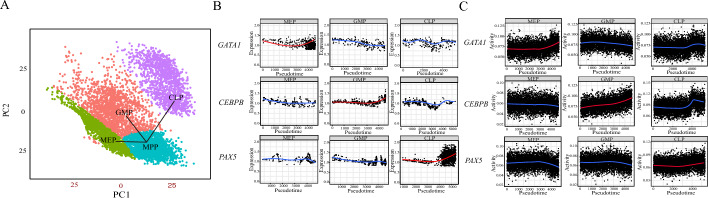
Inference of dynamic changes in cell differentiation by DSSMReg. **A** Three lineages revealed by scRNA-seq data. **B** Gene expression of lineage-specific transcription factors *GATA1*, *CEBPB*, and *PAX5* along the pseudo-time axes of erythroid, lymphoid, and myeloid lineages. Each point represents a cell, with gene expression data smoothed using Locally Estimated Scatterplot Smoothing (LOESS). **C**. Dynamic changes in the activity of the *GATA1*, *CEBPB*, and *PAX5* regulons inferred by DSSMReg along the pseudo-time axis in the CLP, MEP, and GMP lineages

DSSMReg can be integrated with pseudo-time analysis methodologies to facilitate the inference of cellular dynamics during differentiation or developmental events. In our research, we extracted cells from four distinct lineages: CLP, MEP, GMP, and MPP utilizing single-cell transcriptome data derived from human bone marrow hematopoietic stem cells [53]. Leveraging TSCAN [54], we constructed a pseudo-time trajectory that reflects the three primary branches corresponding to CLP, MEP, and GMP (Fig. 6A). The resulting cellular distribution demonstrated three branches aligned with the major lineages of hematopoietic differentiation. Specifically, multipotent progenitors were represented in the lower right quadrant, marked in cyan, while the differentiated cells CLP, MEP, and GMP occupied the terminal ends of these branches.

GATA1, a lineage-specific TF for the erythroid lineage, exhibits an increase in expression levels during the differentiation of erythroid cells. Notably, this expression enhancement is specific to the erythroid lineage, with no equivalent rise detected in the lymphoid or myeloid lineages (Fig. 6B). We utilized DSSMReg to infer the erythroid-specific regulon and calculated AUCell scores for the *GATA1* regulon across individual cells to evaluate its activity. The predicted activity of the *GATA1* regulon corresponded with gene expression data, demonstrating an increase within the erythroid lineage, while no such increase was noted in the lymphoid and myeloid lineages (Fig. 6C). In a similar context, the lineage-specific TF *CEBPB* displayed significant increases in both gene expression and predicted regulon activity within the granulocyte-monocyte lineage, while *PAX5* exhibited lineage-specific increases within the lymphoid lineage (Fig. 6C). The coherence of the regulon activities predicted by DSSMReg with established lineage-specific TF expression patterns [48, 55] underscores the capability of DSSMReg, in conjunction with pseudo-time analysis methods, to effectively infer cellular state transitions at the single-cell level.

## Discussion

We developed DSSMReg, a deep learning method for identifying cell-type-specific regulons and prioritizing their activity. Comparative analyses involving five representative algorithms demonstrated that DSSMReg outperformed the others, achieving the highest AUROC and AUPRC metrics across five distinct cell lines utilizing scRNA-seq data. Moreover, we successfully inferred cell-type-specific regulons from the single-cell transcriptome data of triple-negative breast cancer and human hematopoietic stem cells, underscoring their significant biological relevance. These results indicate that DSSMReg has the potential to advance the understanding of cell differentiation, as well as the gene regulation unique to specific cell types that are implicated in the onset, progression, and suppression of various diseases.

The primary advantage of DSSM in the realm of semantic representation learning is the remarkable capability to integrate disparate information sources. This integration facilitates the mapping of various data types into a shared low-dimensional semantic space. Within this study, we employed DSSM to align the motif and expression data of TFs and TGs into a unified framework, thereby enabling the prediction of regulatory relationships through similarity assessments. The relationships between TFs and TGs are usually characterized by complexity and high dimensionality. Our model captures intricate nonlinear relationships and elucidates latent biological associations that may not be readily observed. Consequently, it enhances the predictive accuracy of the algorithm, rendering it more effective than methodologies that rely exclusively on co-expression or linear models. Furthermore, the shared semantic space created by DSSM enhances the model’s generalization capabilities. Despite the ongoing accumulation of genome-wide DNA binding data for TFs, a considerable portion still lacks ChIP-seq data, and the motifs associated with TF-DNA binding remain incomplete. To address this gap, we integrated single-cell gene expression data to accurately predict regulatory relationships by modeling the interactions among partially observed TFs and their TGs. In the DSSMReg framework, the representations of TFs and TGs coexist within the same semantic space. This arrangement enables the model to identify established regulatory pairs while also predicting analogous TFs and TGs.

The primary limitation of DSSMReg lies in its nature as a supervised deep learning model that requires known gene regulatory relationships to create its training set. Notably, there is a significant disparity in the availability of these known TF-TG regulatory relationships across different species. Human and mouse biological data are relatively abundant, supported by extensive databases such as ENCODE [56] and JASPAR [57]. In contrast, model organisms like *Drosophila*, *yeast*, and *Arabidopsis* have considerably less data, while non-model organisms suffer from an even more pronounced lack of related information. This situation is expected to gradually improve with the continued accumulation of ChIP-seq data. Another strategy to address this challenge is to infer regulatory relationships in closely related species by leveraging existing data. Many TFs demonstrate evolutionary stability, especially those that are crucial for fundamental biological processes. Families of TFs such as *MYB*, *WRKY*, *bHLH*, and *CAMTA* exhibit significant conservation in their sequences, structural features, binding sites, functions, and regulatory mechanisms [58, 59]. This conservation indicates that their regulatory mechanisms and logic are highly similar over extended evolutionary timescales. In earlier studies, researchers developed an adjacency matrix using *Arabidopsis* expression profiles and ChIP-seq data to train a graph neural network. This pretrained model was then transferred to rice, resulting in the successful prediction of numerous regulatory relationships with significant biological relevance [60].

Another limitation is that random sampling may introduce potential false negative labels. We chose this strategy mainly based on the following considerations: first, this strategy is most commonly used in deep learning-based link prediction, and many studies on single-cell gene regulatory network inference have adopted it, demonstrating its effectiveness [16, 61, 62]. Second, the computational efficiency of random sampling enables rapid construction of training sets on large-scale datasets containing thousands of transcription factors and tens of thousands of target genes. Finally, our goal is to establish a model that can be widely used. Random sampling does not rely on specific biological assumptions, thus avoiding bias introduced by such assumptions and ensuring the method’s applicability to multiple samples and species.

We can also consider this issue from the perspective of the sparsity of gene regulatory networks: the proportion of authentic regulatory relationships among all possible TF-TG pairs is tiny; that is, the matrix of all possible TF-TG pairs is highly sparse. In this sparse matrix, the probability of randomly selected TF-TG pairs being authentic regulatory relationships is extremely low, indicating that although random negative sampling strategy may not eliminate false negatives in theory, the vast majority of samples are true negatives. This high proportion of true negatives is also an important reason many models adopt random sampling strategies and perform well.

## Materials and methods

### The network structure of DSSMReg

The structure of DSSMReg consists of two components: the TF network and the TG network, inspired by the deep structured semantic model. The model inputs are the feature vectors of TFs and TGs generated in the previous step. These vectors pass through three fully connected layers with 300, 300, and 128 neurons, respectively, and are transformed into 128-dimensional hidden vectors (Supplementary Table S4). Suppose the vector of a particular TF points to a specific dimension, and the vector of a particular gene is significantly projected on this dimension (i.e., in the same direction). In that case, it suggests that this TF may regulate the gene. We calculate the cosine similarity score *s*_*ij*_ between the hidden vectors of the TF *i* and the TG *j*. This score is then transformed to a value between 0 and 1 using the sigmoid function to assess the likelihood of a regulatory relationship between the TF and the TG (Eqs. 1 and 2).

1 $$ s_{ij} = \cos \left( {{\mathrm{TF}}_{i} ,{\mathrm{TF}}_{j} } \right) = \frac{{{\mathrm{TF}}_{i} *{\mathrm{TF}}_{j} }}{{\left\| {{\mathrm{TF}}_{i} } \right\|\left\| {{\mathrm{TF}}_{j} } \right\|}} $$

2 $$ p_{ij} = {\mathrm{sigmoid}} (s_{ij} ) $$

${\mathbf{TF}}_{i}$, ${\mathbf{TG}}_{j}$ denote the hidden vectors generated by the deep structured semantic model for TF $i$ and TG $j$. Subsequently, binary cross-entropy is employed as the loss function (as shown in Eq. 3), and backpropagation is utilized to update the parameters of both neural networks.

3 $$ BCELoss = - \frac{1}{N}\sum\limits_{i = 1}^{N} {y_{ij} \log (p_{ij} ) + (1 - y_{ij} )\log (1 - p_{ij} )} $$

where $N$ represents the number of training samples, and $y_{ij}$ denotes the true labels of the regulatory relationships between ${\mathbf{TF}}_{i}$ and ${\mathbf{TG}}_{j}$.

### Model training and regulon prediction

The model was built and trained using deep learning software packages PyTorch (1.12.1), TensorFlow (2.11.0), pandas (1.4.3), and numpy (1.23.0). In the model training process, the positive samples are represented by the ground truth regulatory relationships for each dataset, labeled as 1. For each TF, negative samples are randomly selected from genes that do not have regulatory relationships with it. The number of negative samples is aligned with that of the positive samples as much as possible and is labeled as 0. The dataset is subsequently divided into training and testing sets in an 80:20 ratio. After training the deep structured semantic model (Supplementary Table S4), we fix the model parameters of both the TF network and the TG network. The resulting vectors for all TFs and TGs are then processed through their respective networks to compute hidden vectors. The regulatory relationship score between each TF and TG is determined by calculating the cosine similarity of their hidden vectors. This procedure is implemented for each cell type to generate gene vectors, train the model, and predict regulatory relationships, ultimately identifying cell-type-specific regulons. Subsequently, we utilize the AUCell tool [13] to rank the identified regulons, inferring their significance for each cell type. The average AUC value across all cells of a given cell type serves as the score for the cell-type-specific regulons. We compared the performance of DSSMReg with five existing algorithms: DeepRIG [16], MetaSEM [15], DeepSEM [14], GENIE3 [8], and PIDC [12]. We used a paired *t*-test to evaluate the significance of performance differences between DSSMReg and other models.

### Data preparation

We assessed the performance of our model using the BEELINE dataset [23], which comprises five cell types: three mouse cell types and two human cell types. The mouse cell types represent distinct lineages derived from mouse hematopoietic stem cells and include Erythroid, Lymphoid, and Granulocyte–macrophage. We designated them as three separate datasets: mHSC-E, mHSC-L, and mHSC-GM. The mHSC-E dataset consists of 1,071 cells, the mHSC-L dataset contains 847 cells, and the mHSC-GM dataset includes 889 cells. Each dataset retains only the top 1,000 genes ranked by variance. The two human datasets are the human hepatocyte dataset and the human embryonic stem cell dataset, containing 425 cells and 1,019 cells, respectively. For each dataset, we preprocessed the data using Seurat (v4.3.0) [63]. Specifically, we first excluded cells that expressed fewer than 200 genes to ensure that the analyzed cells had sufficient gene expression information. Next, we filtered out genes detected in fewer than three cells to remove those that were not expressed in the majority of cells. Finally, we log-normalized the expression values. For all five datasets, BEELINE provides corresponding ChIP-seq experimentally validated regulatory relationships, which we used as the ground truth for model training and performance evaluation.

We acquired single-cell RNA sequencing data from five untreated TNBC patients through the Tumor Immune Single Cell Hub database [25]. Following a preprocessing method consistent with the previously discussed five datasets, we generated a normalized single-cell expression dataset containing 8,890 cells and 19,356 genes. This dataset encompasses seven cell types: B cells, CD4 T cells, CD8 T cells, endothelial cells, fibroblasts, malignant tumor cells, and monocyte-macrophages. Additionally, we obtained the ground truth regulatory relationships for triple-negative breast cancer from the Cistrome database [26], which includes 154,317 regulatory interactions involving 181 TFs, all validated through ChIP-seq experiments.

We utilized two human bone marrow hematopoietic stem cell scRNA-seq datasets. The first dataset was used to infer the regulons involved in hematopoietic stem cell differentiation, sourced from bone marrow collected from 12 adult femoral heads [47]. After preprocessing with Seurat (v.4.3.0), we obtained expression data for eight types of hematopoietic cells, totaling 13,007 cells, which included 3,659 granulocyte–macrophage progenitor cells, 2,324 hematopoietic stem cells, 2,004 megakaryocyte-erythroid progenitor cells, 1,727 multipotent progenitor cells, 1,346 monocytes, 783 erythroblasts, 739 early B cells, and 486 common lymphoid progenitor cells. The second dataset was used to infer the dynamic changes of cells during differentiation, derived from the single-cell transcriptome of a healthy young donor. After preprocessing, this dataset yielded expression data for four cell lineages, encompassing 6,940 cells, including 3,861 multipotent progenitor cells, 1,487 common lymphoid progenitor cells, 834 megakaryocyte-erythroid progenitor cells, and 767 granulocyte–macrophage progenitor cells. The ground truth labels for both hematopoietic stem cell datasets were obtained from the hTFtarget database [64], which contains the most comprehensive human TF-TG regulatory relationships. We identified 192,435 regulatory relationships among genes that were related to bone marrow tissue and expressed within the datasets.

The CisTarget database contains 34,524 motifs derived from 29 different sources, which include TF motif databases like JASPAR [57] and TRANSFAC [65], as well as experimentally validated ChIP-seq data. The Cluster-Buster tool evaluates the matching degree of each motif within gene regulatory regions, resulting in a score for cis-regulatory modules [66]. This score corresponds to the values of the elements in the gene-motif matrix [13].

### Generation of gene embeddings

DSSMReg generates feature vectors for each TF and TG by integrating both gene expression data and motif information. The expression data is organized in a scRNA-seq matrix, where rows represent genes and columns denote individual cells, with values indicating the normalized expression levels. To derive the feature vector for each gene, we developed a standard autoencoder. This autoencoder consists of two components: an encoder and a decoder. The encoder transforms the high-dimensional expression vector of each gene, aggregated across all cells, into a low-dimensional latent variable. The input to the encoder is the vector of a gene across each cell, represented as, **x** = (*x*_1_, *x*_2_…*x*_n-1_,*x*_n_), Where *n* is the number of cells. The encoder maps the expression vector of each cell in the input layer ${\mathrm{x}} \in {\mathrm{R}}^{{\mathrm{n}}}$ to the embedding layer ${\mathrm{z}} \in {\mathrm{R}}^{{\mathrm{d}}}$, where *d* is the size of the embedding layer. The decoder restores the hidden variable of the hidden layer to an output vector of initial dimension *n*, gradually making the output approach the initial gene expression vector. The reconstructed vector from the decoder network is denoted as **y** = (*y*_1_, *y*_2_…*y*_n−1_, *y*_n_).

4 $$ {\mathrm{y}} = g_{\psi } \left( {f_{\phi } \left( {\mathrm{x}} \right)} \right) $$

where $f_{\phi }$ represents the process of generating the latent vector by the encoder, $g_{\psi }$ represents the process of restoring the original vector by the decoder, and $\phi$ and $\psi$ are the parameters of the encoder and decoder neural networks, respectively. The model’s loss function is defined as:

5 $$ Loss = \min \frac{1}{n}\sum\limits_{i = 1}^{n} {\left\| {x_{i} - y_{i} } \right\|}^{2} $$

The encoder and decoder are composed of fully connected layers with 700 neurons each, using the rectified linear activation function (ReLU) as the activation function. A dropout rate of 0.1 is applied, and the batch size is set to 128. We use the Adam optimizer for model training, with a learning rate of 1.0e − 5, and the size of the embedding layer *d* is set to 50.

Gene2vec treats co-expressed gene pairs as context [67] and employs the skip-gram model from the word2vec algorithm in natural language processing to generate gene embeddings. Inspired by Gene2vec, we utilize the TF-motif annotation files to generate motif feature vectors for transcription factors. Specifically, we use the annotation information of TFs and their corresponding motifs as context, and we apply the word2vec function from the Gensim library (3.8.0) to produce these embeddings [22]. The embedding dimension is set to 50, with other parameters using default values. Through model training, if two TFs share similar “contexts”, meaning they have similar motifs, their motif feature vectors will also be similar. The gene-motif score matrix has genes as rows and motifs as columns, with values representing the cis-regulatory module scores of each gene’s transcriptional start site sequence against the motifs. We constructed another autoencoder to generate 50-dimensional motif feature vectors for each target gene. The input data for the encoder consists of the TGs and their scores for each motif, with the encoder’s structure and parameter settings being the same as those used for encoding the gene expression vectors. Finally, we concatenate the expression vectors and motif vectors of the TFs and TGs to obtain a 100-dimensional feature vector, which is used for training the deep structured semantic model and predicting gene regulatory relationships.

### Inferring cell-type-specific regulons from TNBC cells

We utilized the CellMarker2 database to query marker genes for various cell types in human breast tissue [27] and compared them with the cell type regulatory genes inferred by DSSMReg. We conducted GO enrichment analysis on the *BRCA1* regulon gene set using clusterProfiler (v.4.2.2), applying a significance threshold of *p*-value < 0.05 for determining significant enrichment.

### DSSMReg infers dynamic changes in cell differentiation

We collected single-cell transcriptome data from 6940 human bone marrow hematopoietic stem cells representing four cell lineages: multipotent progenitors, common lymphoid progenitors, granulocyte–macrophage progenitor, and megakaryocyte-erythroid progenitor cells [53]. Given the sparsity of gene expression levels in scRNA-seq data, we employed the statistical imputation method scImpute (v.0.0.09) to fill in the gene expression values [68]. To investigate the dynamic changes in gene expression, we utilized the cluster-based minimum spanning tree (MST) method TSCAN (v.1.32.0) for cell ordering [54]. We first inferred the top 2500 highly variable genes in scRNA-seq, and then inputted the high variable gene expression data into TSCAN for pseudo-time analysis of each cell lineage, obtaining the expression levels of genes in each lineage and the trend of AUCell values over pseudo-time.

## Supplementary Information

Supplementary Material 1

Supplementary Material 2

Supplementary Material 3

Supplementary Material 4

## Data Availability

ScRNA-seq data of Triple-negative breast cancer were downloaded from Gene Expression Omnibus of the National Center for Biotechnology Information (GEO Accession: GSE148673). ScRNA-seq data of human bone marrow hematopoietic stem cells were downloaded from the same database (GEO Accessions: GSE253355 and GSE219015). ScRNA-seq and ChIP-seq data of human embryonic stem cells, human hepatocytes, and mouse hematopoietic stem cells were downloaded from the BEELINE repository at https://zenodo.org/records/3701939. The TF motif annotation and gene motif score matrix were downloaded from the cisTarget database at https://resources.aertslab.org/cistarget/. The TF-TG regulatory relationships for hematopoietic stem cells were obtained from the hTFtarget database at https://guolab.wchscu.cn/hTFtarget/#!/download/. Marker genes for cell types in human breast tissue were obtained from the CellMarker2 database at http://117.50.127.228/CellMarker/CellMarker_download.html. The ground truth regulatory relationships for TNBC cancer are from the Cistrome database at http://cistrome.org/db/#/. The source code, manual, and related materials of DSSMReg are available at https://github.com/YaxinF/DSSMReg.

## Abbreviations

TF: Transcription factor
TG: Target genes
scRNA-seq: Single-cell RNA sequencing
SEM: Structural equation model
GRN: Gene regulatory network
DSSM: Deep structured semantic model
AUROC: Area under receiver operating characteristic curve
AUPRC: Area under precision-recall curve
TNBC: Triple-negative breast cancer
DNNs: Deep neural networks
hESC: Human embryonic stem cells
hHEP: Human hepatocytes cells
mHSCs: Mouse hematopoietic stem cells
HSC: Hematopoietic stem cells
MPP: Multipotent progenitor cells
LMPP: Lymphoid-primed multipotent progenitors
CMP: Common myeloid progenitors
CLP: Common lymphoid progenitors
NK: Natural killer cells
GMP: Granulocyte-monocyte progenitors
MEP: Megakaryocyte-erythroid progenitors
Pre-B: Precursor B cells
MST: Minimum spanning tree
